# Burr hole evacuation of chronic subdural hematoma in general versus local anesthesia: a systematic review and meta-analysis

**DOI:** 10.1007/s00701-025-06475-x

**Published:** 2025-03-08

**Authors:** Clara F. Weber, Kiarash Ferdowssian, Nils Hecht, Peter Vajkoczy, Lars Wessels, Robert Mertens

**Affiliations:** Department of Neurosurgery, Charité – Universitätsmedizin Berlin, Corporate Member of Freie Universität Berlin and Humboldt-Universität zu Berlin, Berlin, Germany

**Keywords:** Chronic subdural hematoma, Burr hole evacuation, Local anesthesia, General anesthesia, Systematic review

## Abstract

**Purpose:**

Chronic subdural hematoma (cSDH) is a highly prevalent condition that frequently requires surgical evacuation. This is typically achieved through burr hole evacuation, which can be performed under either local anesthesia (LA) or general anesthesia (GA). In the present study, we provide a systematic review and meta-analysis to study and compare the safety and efficacy of cSDH evacuation in LA and GA.

**Methods:**

Following the PRISMA guidelines, we screened four databases for studies that compared postoperative outcomes after burr hole evacuation of cSDH in LA versus GA. Baseline characteristics and postoperative outcome data were collected, and risk ratios were calculated for each study as well as pooled across records. Random effect models were applied to continuous data points. Bias was assessed using the MINORS tool.

**Results:**

We identified 22 eligible studies covering 3917 patients in total. LA was associated with decreased risk for complications (p < 0.001), shorter surgery duration (p < 0.001) and hospital stay (p < 0.001). There was no statistically significant association with recurrence rates, postoperative seizure or occurrence of pneumocephalus. In a subanalysis including only data from studies utilizing subdural drainage, results remained largely similar with LA proving advantageous in terms of shorter surgery duration (p < 0.001) and hospital stay (p < 0.001).

**Conclusion:**

LA may serve as a safe alternative to GA for cSDH surgery, associated with fewer postoperative complications and providing benefits regarding shorter hospital stay and surgery duration.

## Introduction

The prevalence of chronic subdural hematoma (cSDH) is increasing [22, 47] and is projected to constitute the most common cranial neurosurgical condition by 2030 [4]. The occurrence of cSDH has historically been linked to mild head trauma, which can cause bleeding into the subdural space due to rupture of bridging veins [16]. However, a revised understanding of its etiology encompasses multiple contributing factors that extend beyond mechanical injury. CSDH originates from an interplay of sustained inflammatory processes [27, 28] and age-related alterations in angiogenesis and fibrinolysis [24, 42], ultimately impacting dural border cells [14]. Additionally, the pro-angiogenic and proliferative effects of inflammatory cells perpetuate a cycle of capillary formation and fragility, thereby sustaining the chronic hematoma [14]. CSDH typically presents with delayed-onset, progressive symptoms such as confusion, headache, dizziness, and a decreased level of consciousness [16, 19, 31]. Depending on the laterality of the hematoma, hemisymptoms, such as dysphasia and hemiparesis, are also common [19]. Elderly patients are particularly susceptible to cSDH due to age-related brain atrophy and changes in inflammation response and angiogenesis, further promoting cSDH progression [14, 23, 59]. Common treatment strategies involve surgical hematoma evacuation to reduce the mass effect. This can be achieved through small craniotomies [37], drainage using hollow screws (twist-drill craniostomy) [32], and, most commonly, classic burr hole evacuation [31, 38]. The latter involves trepanning the skull and flushing of the subdural space to remove the hematoma and additional liquid residues [12], while the optimal placement of the burr hole remains a matter of ongoing debate [60]. While specific protocols differ between institutions, e.g. regarding burr hole location and drainage insertion, burr hole trepanation for cSDH surgery is widely recognized as safe and constitutes one of the most common cranial neurosurgical procedures [12, 19, 31].

Surgical cSDH evacuation is typically conducted under general anesthesia [12]. As this pathology preferentially affects elderly patients, who are at greater risk of periprocedural complications such as delayed awakening, postoperative delirium and confusion [49, 52], cSDH surgery is associated with non-negligible perioperative risks. Thus, the feasibility and safety of cSDH surgery under local anesthesia (LA) has been discussed to moderate and potentially circumvent postoperative complications. General anesthesia (GA) usually involves the administration of several pharmacological agents and endotracheal intubation [15]. LA however is achieved through the infiltration of local anesthetics such as lidocaine into the skin and subcutaneous tissues [53], whereas the bone and meninges do not require anesthesia [29]. An overall refinement of the rationale behind cSDH surgery in LA is the potential favorable risk profile for perioperative risk and post-anesthesia complications, especially given the vulnerability of the cSDH patient population towards post-GA complications [3, 63].

In an effort to summarize existing evidence on anesthesia techniques for cSDH surgery, recent systematic reviews have aggregated literature on postsurgical outcomes, overall indicating favorable risk profiles and safety of cSDH evacuation in local anesthesia [1, 34, 35]. However, critiques of these studies have highlighted that varying surgical techniques can exert distinct influences on outcome measurements [41]. Different surgical approaches, including burr hole evacuation, craniotomy and twist-drill craniostomy, were grouped together, despite significant differences in their invasiveness and the extent of required tissue manipulation [6, 31]. This methodological heterogeneity likely confounds postsurgical outcomes. Additionally, previous reviews have been criticized for potential reporting biases [41], highlighting the need for future reviews specifically addressing these limitations.

In this systematic review and meta-analysis, we aimed to expand upon the existing literature on anesthetic approaches in cSDH surgery, with a specific focus on burr hole trepanation. By contextualizing our findings with previous reports, we sought to strengthen the evidence base for reliable and safe neurosurgical treatment strategies for cSDH.

## Methods and materials

Reporting followed the Preferred Reporting Items for Systematic Reviews and Meta-Analyses (PRISMA) statement [45]. We screened four major databases (Cochrane, NCBI PubMed, Ovid MEDLINE, Scopus) in July 2024 using the following combination of Medical Subject Header (MeSH) Terms: “Hematoma, Subdural” AND (“Anesthesia, Local” OR “Anesthesia, General”).

Inclusion criteria were defined as (i) randomized control trials, case–control and cohort studies that (ii) reported postsurgical outcomes, (iii) specifically for burr hole evacuation in GA and/or LA. Exclusion criteria encompassed (i) non-human studies, (ii) studies covering any surgical approaches other than burr hole evacuation, (iii) studies that were not published in peer-reviewed journals, or (iv) written in non-English language. To maximize the similarity of surgical approaches between studies, we further excluded all single-arm studies that did not report subdural drainage insertion. Within comparative studies, we included all studies irrespective of drainage use in the main analysis and conducted follow-up confirmative testing in a subgroup consisting exclusively of studies with confirmed subdural drainage.

Abstracts were extracted for all initially identified studies and screened for eligibility under the given inclusion and exclusion criteria, followed by text screening of those articles that were not eliminated in the abstract review. The screening and extraction were conducted independently by a medical graduate (CFW) and a neurosurgical resident physician (RM). Conflicts were discussed and resolved through consensus, supervised by a board-certified attending neurosurgeon (LW). Studies were segregated into single-arm studies for postsurgical outcomes of cSDH burr hole surgery in GA or LA, as well as comparative studies. Subsequently, demographic data and baseline characteristics involving age, sex and American Society of Anesthesiologists’ (ASA) perioperative risk scores distribution as well as clinical data about the initial presentation, postoperative outcomes, complications and anesthesiologic and surgical protocols were extracted from all studies.

### Statistical analysis

Risk ratios (RR) were calculated for postsurgical outcome metrics and significance was assessed using Fisher’s exact test [17]. For the purpose of this analysis, the GA group was designated as the control group (reference), while the LA group was defined as the intervention group. Risk ratios were reported with GA as reference, meaning that an RR greater than 1 indicates a higher risk in the LA group compared to the GA group, and an RR less than 1 indicates a lower risk in the LA group. Furthermore, continuous variables were assessed fitting random effects models [10]. Studies were excluded from synthesis if they did not report on the measure in question. Statistical significance was assumed at p < 0.05, and p-values were adjusted for multiple comparisons using Benjamini and Hochberg’s false discovery rate (FDR) correction [5]. Data analysis was conducted in R statistical software version 4.3.1 [48] using the *metafor* package [57]. Heterogeneity across studies was assessed using I^2^,. This metric indicates the nonrandom variation among studies, with I^2^ > 50% indicating considerable heterogeneity across studies. Risk of bias was assessed using the risk methodological index for non-randomized studies (MINORS) [51]. Briefly, this tool aggregates eight methodological items for non-comparative studies, with an additional four items for comparative reports that are rated with zero (not reported), one (reported but inadequate) or two (reported and adequate) points. Comparative and non-comparative studies can thus reach a maximum score of 24 and 16 points respectively, and a score of ≥ 12 (comparative studies) or ≥ 8 points (non-comparative studies) is considered satisfactory [51].

## Results

**Fig. 1:**
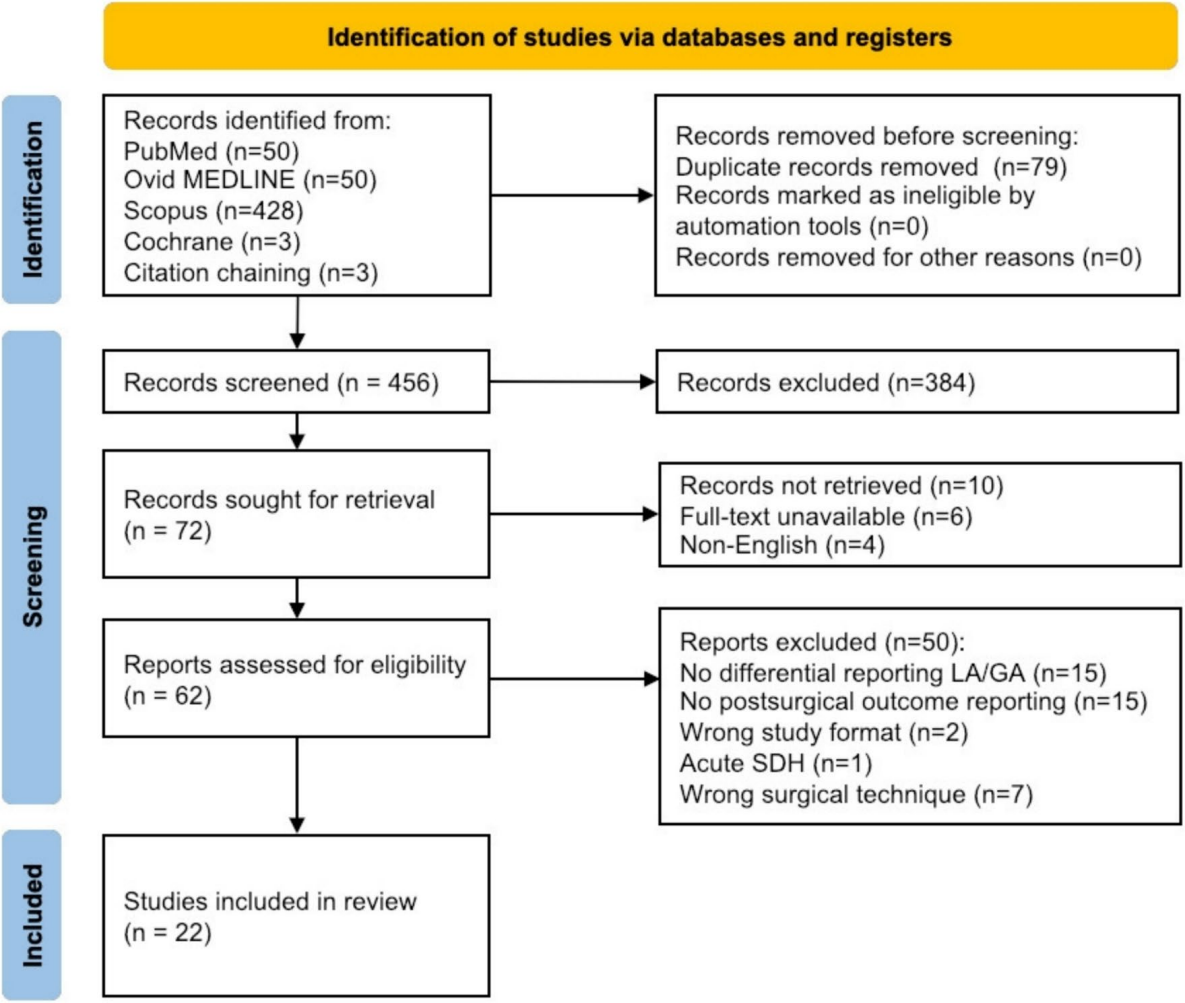
Flowchart of study selection. FDR = false discovery rate-corrected, GA = general anesthesia, LA = local anesthesia

The initial database screening yielded a total of n = 455 articles (NCBI PubMed: 50, Ovid MEDLINE: 50, Scopus: 428, Cochrane Library: 3, citation chaining: 3; 79 duplicates). Studies that did not report drainage use or did not report postoperative outcome measures were excluded. The detailed workflow reflecting the screening and exclusion of articles is depicted in Fig. 1**.** We finally included 9 comparative articles covering both LA and GA [3, 6, 7, 13, 21, 54, 58, 61, 63], as well as 8 single-arm LA studies [11, 18, 20, 25, 39, 44, 50, 56] and 5 GA studies [8, 9, 26, 40, 62]. Altogether, the finally selected articles covered aggregated data of 3917 cases, of which 1921 patients underwent surgery in LA and 1996 patients received GA.

Baseline characteristics of the sample are presented in Table 1**.** Overall, the majority of patients were male and in their 5th to 7th decade, though age ranges varied widely and included young adults. Conversely, one study by Tabuchi et al*.* focused on nonagenarians [56]. Across records, most patients, 73.1 ± 10.3%, were male. Most comparative studies were based on retrospective chart reviews, whereas one study by Surve et al*.* presented a randomized trial [54]. Most included reports considered both uni- and bilateral cSDH in their analysis. Preoperative Glasgow Coma Scale (GCS) scores, while reported heterogeneously, were largely similar between LA and GA groups, and predominantly showed only mild impairments (largely > 13; Table 1). Preanesthetic risk evaluation according to the American Society of Anesthesiologists (ASA) classification ranged between I and IV across studies, with most studies indicating slightly higher scores in the LA groups. Where reported, range and mean ASA scores are displayed in Table 1.

**Table 1:** Baseline characteristics of all included studies

	Author	Acquisition years, location, n (sites)	n (GA/LA)	% male (GA/LA)	Age GA/LA mean ± SD [years]	% unilateral, % left (GA/LA)	Preoperative GCS mean ± SD (GA/LA)	ASA range, mean (GA/LA)
LA/GA comparative	Ashry et al., 2022	2018–2020, Egypt (1)	45 (23/22)	74/76	73.2 ± 1.7/74.3 ± 3.5	< 100%, na	12.55 ± 0.7/12.96 ± 1.48	I-IV, 2.34/2.86
	Blaauw et al., 2020	2005–2019, Netherlands (3)	923 (314/609)	74/76	73 ± 11.4/74 ± 10.7	83.6%, na	na	na
	Certo et al., 2019	na, Italy (1)	30 (15/15)	66.7/60	77.1 (57–87)/76.4 (61–91)	na, na	na	na
	Durdağ et al., 2013	2008–2012, Turkey (1)	26 (16/10)	73.3/80	58.6 ± 16.1/63.7 ± 16.1	62.6%, na	na	na
	Havryliv et al., 2024	2020–2022, Ukraine (1)	67 (44/23)	73/83	58.6 ± 16.1/63.7 ± 16.1	30%/13%, na	13%/30.4% GCS < 15	na
	Surve et al., 2017	2013, India (1)	72 (34/38)	91.2/89.5	58.79 ± 14.97/57.63 ± 15.08	na, na	13.85 ± 1.76/ 14.03 ± 1.48	I-IV/I-III, 1.53/1.45
	Tuncer et al., 2019	2016–2018, Turkey (1)	27 (11/16)	45.5/62.5	LA and GA: 74.67 ± 15.21	na, na	13.64 ± 0.81/ 13.69 ± 0.79	II-III/II-IV, 2.73/3.12
	Wong et al., 2022	2006–2020/2018–2020, Malaysia/UK (2)	257 (127/130)	80.3/76.2	69.5 ± 12.4/66.8 ± 14.3	81/67%, 45.7/38.5%	73.2%/88.5% GCS > 12, 8.7%/3.5% GCS < 9	I-II
	Zhuang et al., 2022	2013–2018, China (1)	105 (54/51)	83.3/90.2	66.63 ± 12.14/62.25 ± 11.54	na, 51.9/62.7%	14.89 ± 0.42/14.9 ± 0.36	na
GA	Choi et al., 2016	2007–2016, South Korea (1)	502	67.9	67 ± 13	< 100%, na	na	na
	Choi et al., 2020	2011–2016, South Korea (1)	286	71.3	69.4 ± 13.1	83.4%, na	62.6% GCS > 12, 1.7% GCS < 9	na
	Hwang et al., 2022	2015–2019, South Korea (1)	203	79.7	71.4 ± 12.7	82.3%, na	88.2% GCS > 12, 2.5% GCS < 9	na
	Morales-Gómez et al., 2020	2014–2018, Mexico (1)	155	81.9	65.9 ± 16.6	80%, 43.9%	83.9% GCS > 12, 7.1% GCS < 9	na
	Yan et al., 2023	2010–2022, China (2)	212	82.08	68.71 ± 13.01	na, na	na	na
LA	Dobran et al., 2022	2015–2019, Italy (1)	104	69.35	77.4 ± 14.3	na, na	na	na
	Guzel et al., 2008	2001–2006, Turkey (1)	20	na	60.9 (18–93)	na, na	na	I-IV, 2.95
	Hashimoto et al., 2023	2005–2021, Japan (1)	223	73.5	76.9 [49–95]	84%, na	42% GCS < 15	na
	Honda et al., 2021	2013–2020, Japan (1)	194	74.2	79.6 ± 9.2	75%, 45%	na	na
	Mersha et al., 2020	2012–2015, Ethiopia (1)	195	70.3	57.63	< 100%, na	na	na
	Ozdol et al., 2024	2008–2020, Turkey (1)	201	na	64 ± 18	73%, na	na	na
	Sauvigny et al., 2024	2017–2021, Germany (1)	50	62	72.9 ± 10.7	na, na	na	na
	Tabuchi et al., 2014	2007–2013, Japan (1)	20	55	92.6 ± 1.9	83.3%, 41.7%	na	na

*ASA *American Society of Anesthesiologists perioperative risk assessment, *LA *local anesthesia, *GA* general anesthesia, *GCS* Glasgow Coma Scale score, *SD *standard deviation

### Anesthesiologic technique and periprocedural measures

Anesthesiologic protocols described local infiltration of the skin and subcutaneous tissues with sodium channel blockers, most commonly lidocaine and bupivacaine. Many studies reported additional sedation with benzodiazepines [3, 21, 39, 61] or dexmedetomidine [54], some with additional intravenous administration of analgesics [18, 50]. GA was achieved using standard, propofol- [8, 21, 63] or thiopentone- [54] based intravenous anesthesia together with intravenous administration of fentanyl for additional pain relief, alongside endotracheal intubation. Anesthesiologic specifications are listed in Table 2**.**

**Table 2:** Procedural and anesthesiologic baseline characteristics of all included studies

	Author	Mortality (LA/GA)	Antithrombotic therapy (LA/GA)	ASA	Preoperative GCS (LA/GA)	Anesthesiologic specifications (LA/GA)	Complication types (LA/GA)
GA/LA comparative	Ashry et al., 2022	na/na	na/na	I-IV, mean 2.86/2.34	12.96 ± 1.48/2.55 ± 0.7	0.5% bupivacaine + 2% lidocaine + propofol/midazolam selectively for irritable patients	Tension pneumocephalus (4.5%/8.7%), recollection (4.5%/17.3%), seizures (4.5%/8.7%), uncontrolled hypertension (4.5%/4.3%), diabetic coma (0%/4.3%), myocardial infarction (0%/8.7%), wound infection (4.5%/0%)
	Blaauw et al., 2020	4.3%/3.2%	Overall: 24% antiplatelet, 32% oral anticoagulants	na	Overall median: 15	na	Delirium (29%), Pneumocephalus (18%), Empyema (13%), seizures (12%), systemic infection (7%), thrombembolic incident (3%), other (aphasia, CSF leak, SAH – 8%). Complications not reported for LA and GA separately
	Certo et al., 2019	na/na	na/na	na	na	na	Recurrence (0/6.7%), Worsening of preexisting neurodegenerative disease (0/20%), Parkinson-associated gait disturbance (6.7/0%)
	Durdağ et al., 2013	0%/6.3%	30%/18.8%*	na	na	na	na
	Havryliv et al., 2024	na/na	30.4%/9.1%*	II-IV/II-III	30.4%/13% < 14	Diazepam, 2% lidocaine (6–10 ml)/propofol, fentanyl, atracurium	Pneumocephalus (13%/27%)
	Surve et al., 2017	0%/2.9%	na/na	I-III, mean 1.45/I-IV, mean 1.53	14.03 ± 1.48/13.85 ± 1.76	2 ml 0.5% bupivacaine, 2 ml 0.5% lignocaine with adrenaline, dexmedetomidine 1 µg/kg bolus + 0.5 µg/kg/h maintenance	Bradycardia (2.6/2.9%), postoperative snoring, stridor or throat pain (0/14.7%), restlessness and agitation (0/2.9%), pneumocephalus (2.6/0%)
	Tuncer et al., 2019	na/na	na/na	3.13 ± 0.62 (II-IV)/2.73 ± 0.47 (II-III)	13.69 ± 0.79/13.64 ± 0.81	na	na
	Wong et al., 2022	0%/3.9%	29.2%/22.8%*	I-II	88.5%/73.2% 13–15, 3.5/8.7% < 9	GA: fentanyl 1–2 µg/kg + propofol 1–2 mg/kg; LA: midazolam 0.02 mg/kg + 0.5 mg/kg pethidine	Pneumonia (0%/6.3%), cardiac (0%/3.1%), sepsis (0%/2.4%), stroke (0%/0.8%), AKI (0%/0.8%), electrolyte imbalance (0%/0.8%), ICB (0%/0.8%), hypotension (0%/0.8%), seizure (2.3%/0%), hemianopia (0%/0.8%), subdural empyema (0%/0.8%), wound infection (1.6%/0%), pseudomeningocele (0%/0.8%), subdural infection (0%/0.8%)
	Zhuang et al., 2022	na/na	0%/0%	na	14.9 ± 0.36/14.89 ± 0.42	1% lidocaine s.c./midazolam 2 mg, 1%propofol 7 ml, fentanyl 0.1 mg i.v., cisatrucrium 10 mg + 1%propofol 1.5 µg/kg/min + remifentanyl 15 µg/kg/min	Pneumonia (0%/5.6%), restlessness (0%/7.4%), vomiting (3.9%/13%), sore throat (0%/3.7%), delayed awakening (0%/7.4%), epilepsy (2%/1.9%), poor wound healing (2%/0%), dyspnea (0%/11.1%), intracranial infection (0%/1.9%)
GA	Choi et al., 2016	10.8%	na	na	na	Fentanyl, thiopentone, vecuronium, ETT	Recurrence (7.8%), stroke (0.8%), hydrocephalus (0.8%), intracranial abscess (0.8%)
	Choi et al., 2020	na	10.5% antiplatelet, 2.1% anticoagulant	na	15–14: 74.3%, 13–9: 15.7%, 8–3 10%	na	Recurrence (21.3%)
	Hwang et al., 2022	3.4%	24.1 antiplatelet (4.4% dual, 19.7% single), 5% anticoagulant (2% VKA, 3% NOAC)	na	13–15: 88.2% 9–12: 9.4%, 3–8: 2.5%	na	Bleeding at surgical site (3%) or remote site (0.5%), pneumonia (4.4%), UTI (0.5%)
	Morales-Gómez et al., 2020	6.4%	1.9% antiplatelet, 3.2% anticoagulant		15–13: 83.9%, 12–9: 9%, < 9 7.1%	na	Pneumonia (6.5%), seizures (5.8%), arrhythmia (1.3%), acute myocardial infarction (1.3%), renal failure (0.6%), reoperation (10.3%), recurrence (1.3%), aSDH (2.6%), subdural empyema 1.3%, parenchymal hematoma (0.6%), abscess (0.6%), epidural hematoma (0.6%)
	Yan et al., 2023	na	na	na	na	na	Recurrence (7.07%)
LA	Dobran et al., 2022	na	0%	na	na	na	Recurrence (20.8%)
	Guzel et al., 2008	0	na	I-IV, mean 2.95	na	2–3 ml 0.5% bupivacain, i.v. midazolam 0.03 mg/kg for induction + continuous 0.015–0.06 mg/kg/hr, fentanyl 0.5 mg/kg I.v. bolus + 0.25 mg/kg/min	Residual effusion (10%)
	Hashimoto et al., 2023	na	20% antiplatelet, 13% anticoagulant, 3% both	na	42% < 14	na	Hemiplegia (3%), Gait disturbance (10%)
	Honda et al., 2021	na	22.2%*	na	na	na	Recurrence (11.3%)
	Mersha et al., 2020	2.1%	na	na	na	10 cc 2% lidocaine with adrenaline, i.v. valium or pethidine if necessary	Recollection (6.6%)
	Ozdol et al., 2024	na	60%	na	na	na	Recurrence (10%), Pneumocephalus (54%), seizures (18%)
	Sauvigny et al., 2024	na	28% antiplatelet, 22% anticoagulant (10% VKA, 12% DOAC)	na	na	Equal parts lidocaine 1%/ropivacaine 2 mg/ml, intravenous analgesics if deemed necessary	aSDH (4%), recurrence requiring reoperation (12%)
	Tabuchi et al., 2014	0%	25%*	na	na	na	Transient postoperative restlessness, falls

In fields marked by an asterisk (*), original studies did not discriminate between antiplatelet and/or anticoagulant use. *AKI*  acute kidney injury, *aSDH* acute subdural hematoma, *DOAC *direct oral anticoagulants,* ETT *endotracheal intubation, *ICB *intracranial bleed, *GA* general anesthesia, *LA *local anesthesia, *VKA* vitamin K antagonist

Moreover, we extracted information about preexisting medical conditions and medication history. Antiplatelet and anticoagulant drug intake reached between 5.2 and 60%, with two studies by Dobran et al*.* [11] and Zhuang et al*.* [63] excluding patients under antithrombotic therapy (Table 2). Previous medical history included arterial hypertension, diabetes mellitus, respiratory and psychiatric conditions among others (Table 3).

**Table 3:** Preexisting medical conditions in % per study cohort

	Author	Hypertension (LA/GA)	CV (LA/GA)	Hepatic (LA/GA)	Diabetes (LA/GA)	Psychiatric (LA/GA)	Respiratory (LA/GA)	Infectious (LA/GA)	Neoplasm (LA/GA)	Hematologic (LA/GA)	Kidney (LA/GA)
GA/LA comparative	Ashry et al., 2022	45.5%/26.1%	13.6%/17.4%	13.6%/21.7%	27.3%/60.9%	na	22.7%/17.4%	na	na	na	18.2%/26.1%
	Blaauw et al., 2020	na	1.3%/0%	0.7%/0%	na	na	0.5%/0%	na	na	0.2%/0%	na
	Certo et al., 2019	na	na	na	30/25%	na	na	na	na	na	na
	Durdag et al., 2013	50/18.8%	na	na	30%/25%	na	na	na	na	na	10%/0%
	Havryliv et al., 2024	na	95.7%/93.2%	8.7%/0%	13%/6.8%	4.3%/4.5%	8.7%/2.3%	na	na	0%/2.3%	na
	Surve et al., 2017	na	na	na	na	na	na	na	na	na	na
	Tuncer et al., 2019	na	na	na	na	na	na	na	na	na	na
	Wong et al., 2022	31.5%/63.8%	16.2%/29.1%	na	11.5%/22.8%	na	1.5%/7.9%	3.8%/4.7%	0%/1.6%	2.3%/0%	2.3%/1.6%
	Zhuang et al., 2022	na	na	na	9.8%/11.1%	na	3.9%/1.9%	na	na	na	2%/0%
GA	Choi et al., 2016	na	na	na	na	na	na	na	na	na	na
	Choi et al., 2020	41.6%	na	na	15.4%	na	na	na	na	na	na
	Hwang et al., 2022	44.3%	16.7%	0.5%	26.6%	na	1%	na	6.4%	na	3.4%
	Morales-Gómez et al., 2020	27.7%	1.9%	na	25.2%	na	0.6%	na	na	na	na
	Yan et al., 2023	na	na	na	na	na	na	na	na	na	na
LA	Dobran et al., 2022	na	na	na	na	na	na	na	na	na	na
	Guzel et al., 2008	35%	40%	20%	5%	na	15%	na	na	5%	na
	Hashimoto et al., 2023	na	na	na	na	na	na	na	na	na	na
	Honda et al., 2021	55.1%	na	na	23.7%	na	na	na	na	na	6.2%
	Mersha et al., 2020	na	na	na	na	na	na	na	na	na	na
	Ozdol et al., 2024	na	40%	na	30%	na	6.6%	na	na	na	na
	Sauvigny et al., 2024	na	na	na	na	na	na	na	na	na	na
	Tabuchi et al., 2014	na	na	na	na	41.7%	na	na	na	na	na

*CV *cardiovascular diseases, *GA *general anesthesia, *LA* local anesthesia

In comparative studies, LA was associated with significantly lower operative times (mean difference (MD) −21.33 min; 95% confidence interval (CI) −33.45, −9.20; p_FDR_ < 0.001). In single-arm studies, mean operative time was 37.3 ± 2.8 min and 45.0 ± 8.4 min in LA and GA studies, respectively.

### Postoperative outcome measures

**Fig. 2:**
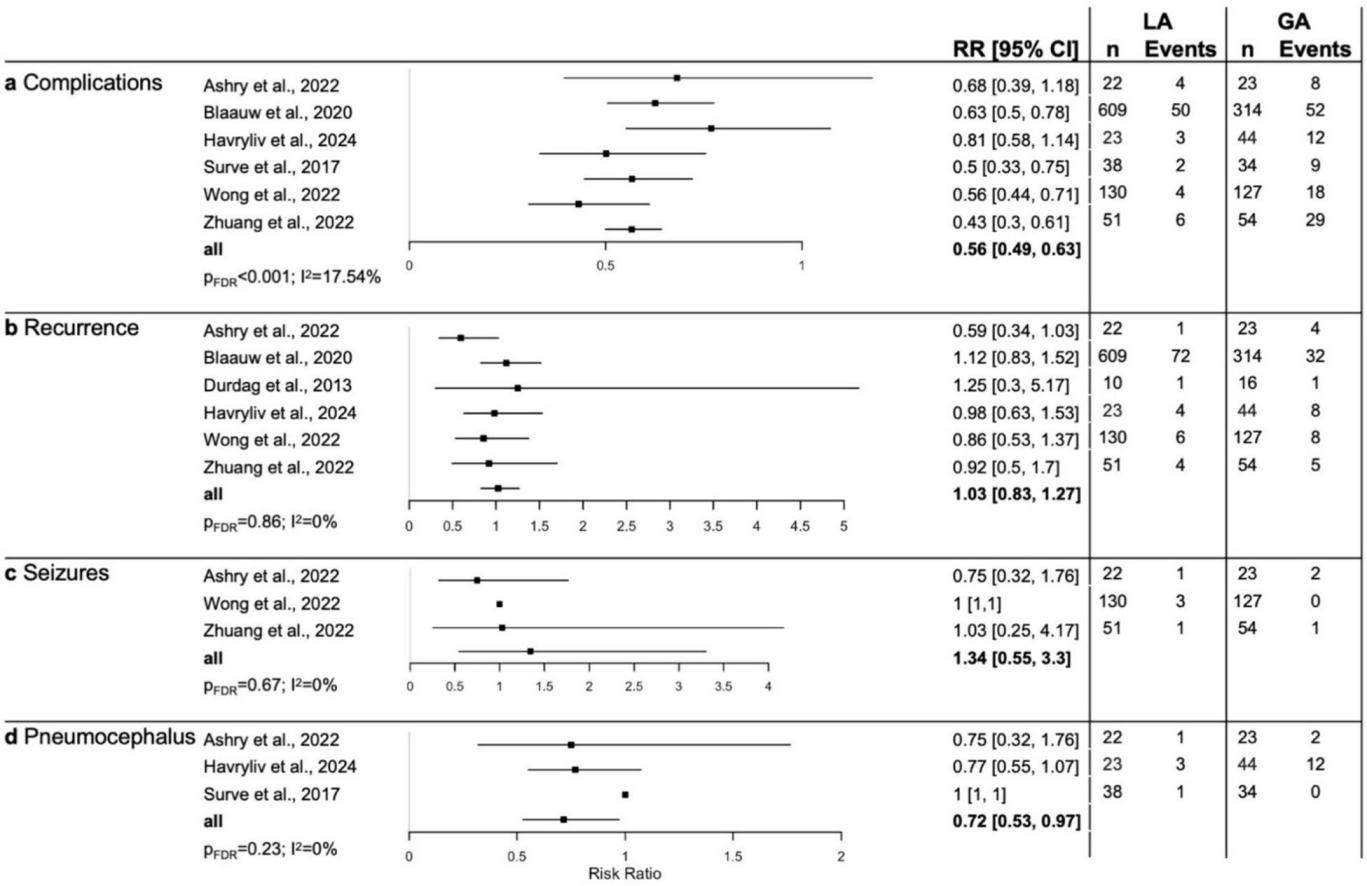
Risk ratios from comparative studies for **a** overall postsurgical complications, **b** recurrence of cSDH, **c** seizures and **d** pneumocephalus. CI = confidence interval, FDR = false discovery rate-corrected, GA = general anesthesia, LA = local anesthesia, RR = risk ratio

**Fig. 3:**
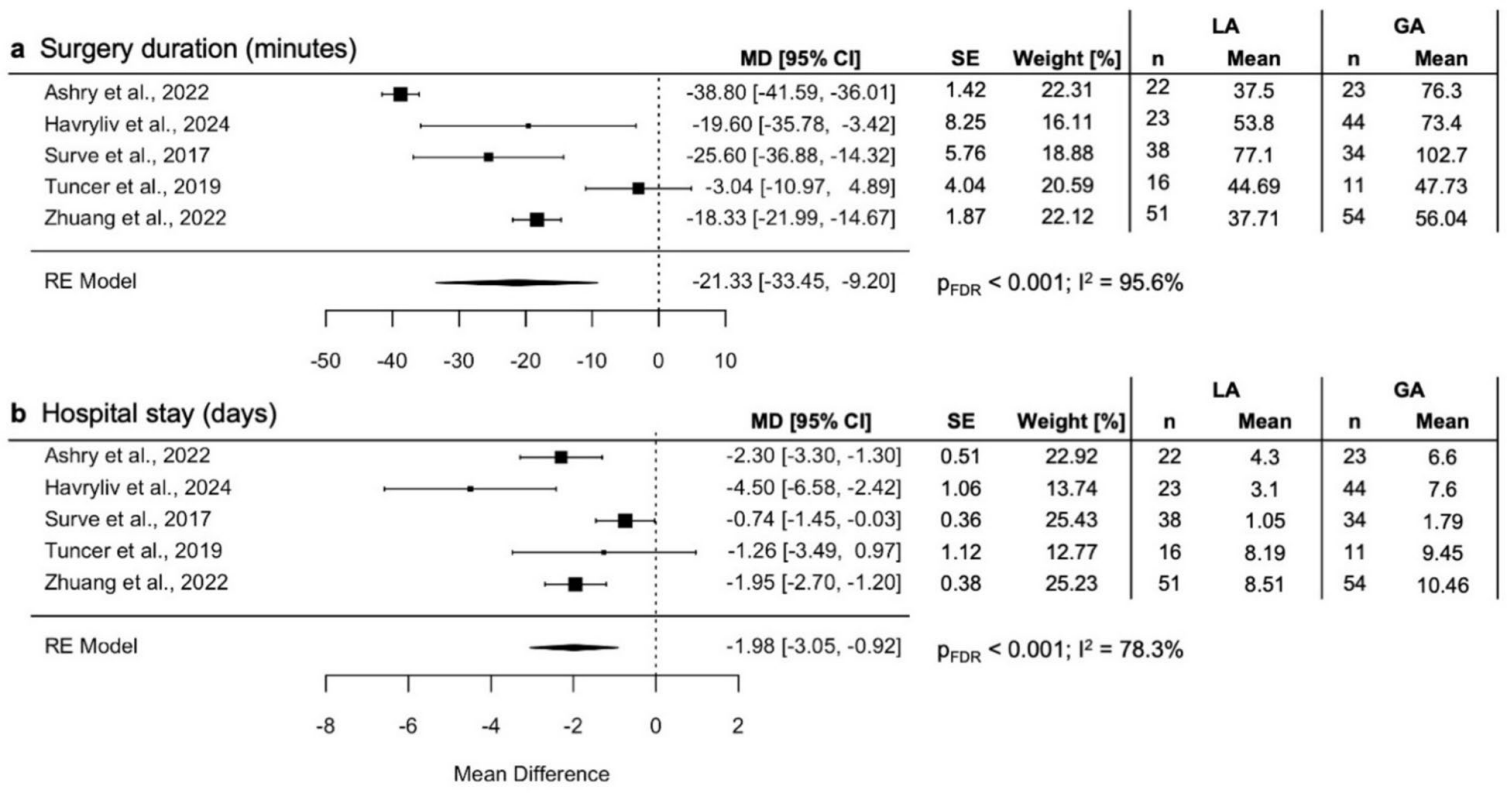
Mean difference from random effect models analyzing comparative studies for **a** surgery duration (hours) and **b** length of hospital stay (days). FDR = false discovery rate-corrected, GA = general anesthesia, LA = local anesthesia, MD = mean difference, RE = random effect model, SE = standard error

In comparative studies, LA was associated with a lower rate of postoperative complications (risk ratio (RR) 0.56; 95% CI: 0.49, 0.63; p_FDR_ < 0.001). Studies named various postoperative complications, including cardiovascular events, dyspnea and pneumonia, as well as surgical complications including wound infection and pneumocephalus. To account for potentially biased reporting in comparative studies, we compared complications by anesthesia type with those reported in single-arm studies. Postoperative complication types and frequencies were largely similar when comparing study arms and single-arm studies for each anesthesia type, respectively, as summarized in Table 2. The risk ratios per comparative study are depicted in Fig. 2**.** In detail, recurrence rates were not significantly favorable towards either group (RR 1.03; 95% CI 0.83, 1.27; p_FDR_ = 0.86). Similarly, there was no clear advantage detectable in wound infection rates (RR 2.51; 95% CI 0.43, 14.53; p_FDR_ = 0.37) or postoperative seizures (RR 1.34; 95% CI 0.55, 3.3; p_FDR_ = 0.67). Of note, LA was associated with lower, although not statistically significant reduced risk for pneumocephalus (RR 0.72; 95% CI 0.53, 0.97; p_FDR_ = 0.23). Additionally, postoperative length of hospital stay was significantly lower in the LA group (MD −1.98 days; 95% CI −3.05, −0.92; p_FDR_ < 0.001), as well as duration of surgery (MD −21.33 min; 95% CI −33.45, −9.20; p_FDR_ < 0.001) (Fig. 3). Mortality rates did not favor LA or GA as reported by Blaauw et al*.* [6] (RR 1.23; 95% CI 0.72, 2.11; p_FDR_ = 0.48), while three additional comparative studies by Durdağ et al*.*, Surve et al*.,* and Wong et al*.* found mortality only in the GA group **(**Table 2**)**.

### Subanalysis regarding confirmed subdural drainage

**Fig. 4:**
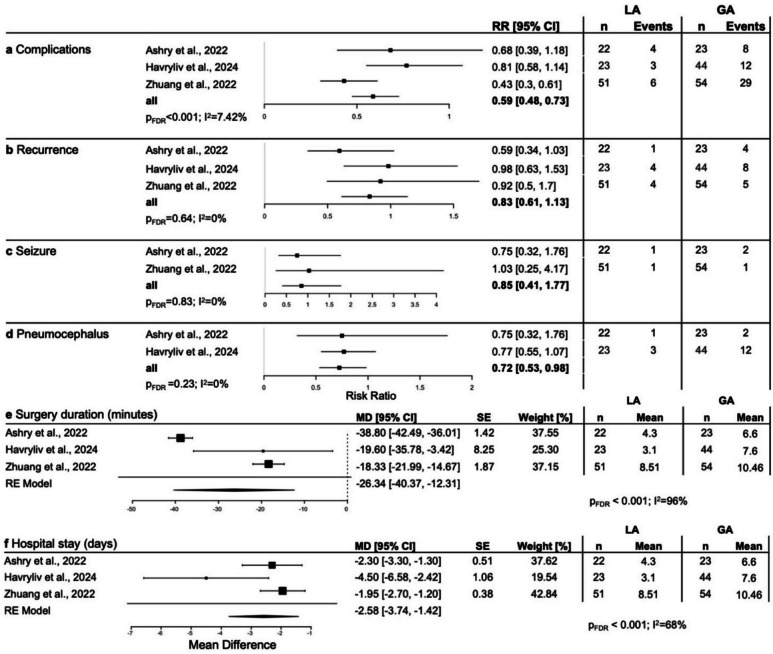
Risk ratios from comparative studies for **a** overall postsurgical complications, **b** recurrence of cSDH, **c** seizures and **d** pneumocephalus and mean difference from random effect models for **e** surgery duration (hours) and **f** length of hospital stay (days) in a subset of studies with confirmed use of postsurgical subdural drainage. FDR = false discovery rate-corrected, GA = general anesthesia, MD = mean difference, LA = local anesthesia, RE = random effect model, RR = risk ratio, SE = standard error

To ensure maximum comparability across studies, we conducted a subanalysis involving only studies that covered burr hole trepanation with confirmed insertion of a subdural drainage. All single-arm studies [8, 9, 11, 18, 20, 25, 26, 39, 40, 44, 50, 56, 62] as well as comparative studies by Ashry et al*.* [3]*,* Havryliv et al*.* [21] and Zhuang et al*.* [63] confirmed use of subdural drainage systems. While most findings showed a similar direction of favorability, measures did not reach statistical significance. Risk ratios for overall surgical complications (RR: 0.59; 95% CI: 0.48, 0.73; p_FDR_ < 0.001), recurrence (RR: 0.83; 95% CI: 0.61, 1.13; p_FDR_ = 0.64), postoperative seizures (RR: 0.85; 95% CI: 0.41, 1.77; p_FDR_ = 0.83), and pneumocephalus (RR 0.72; 95% CI: 0.53, 0.98; p_FDR_ = 0.23) are shown in Fig. 4a-d. Surgery duration and hospital stay remained favorably decreased in the LA group (MD −26.34 min; 95% CI: −40.37, −12.31; p_FDR_ < 0.001, and MD −2.58 days; 95% CI: −3.74, −1.42; p_FDR_ < 0.001, respectively).

### Bias assessment

Bias according to the MINORS assessment is shown in Table 4**,** with mean scores of 18.8 ± 2.0 of maximum 24 points in comparative studies, and 10.6 ± 1.2 of 16 and 11.5 ± 1.7 of 16 points for single-arm LA and GA studies, respectively. All studies exceeded minimum satisfaction thresholds of 12 or 8 points in comparative and non-comparative studies respectively. Most notable deficits across groups were noted in the prospective study size calculation and data collection categories, as well as lack of follow-up reporting. Regarding heterogeneity, I^2^ measures were low in risk ratio calculations for complications, recurrence, postoperative seizures and pneumocephalus, thus indicating good generalizability of these results. In surgery duration and hospital stay assessments however, we saw high heterogeneity with I^2^ ranging from 78.3 to 95.6% in random effects models.

**Table 4:** Risk of bias assessment according to the MINORS tool

Study	Clearly stated aim	Consecutive patient inclusion	Prospective data collection	Appropriate endpoints	Unbiased assessment of endpoint	Appropriate follow-up period	Loss to follow up < 5%	Prospective study size calculation	Control group adequacy	Contemporary groups	Baseline equivalence	Adequate statistical analyses
Ashry et al., 2022	2	2	1	2	2	2	2	0	2	2	2	2
Blaauw et al., 2020	2	2	1	2	2	2	0	0	2	2	2	2
Certo et al., 2019	2	1	2	2	2	2	2	1	2	2	2	2
Durdağ et al., 2013	2	0	1	2	2	2	0	0	2	2	2	2
Havryliv et al., 2024	2	2	1	2	2	2	0	0	2	2	2	2
Surve et al., 2017	2	2	2	2	2	2	1	0	2	2	2	2
Tuncer et al., 2019	2	0	1	2	2	2	0	0	2	2	2	2
Wong et al., 2022	2	0	1	2	2	2	0	0	2	2	2	2
Zhuang et al., 2022	2	0	1	2	2	2	0	0	2	2	2	2
Choi et al., 2016	2	2	1	2	2	2	2	0	na	na	na	na
Choi et al., 2020	2	2	1	2	2	2	1	0	na	na	na	na
Hwang et al., 2022	2	2	1	2	2	2	0	0	na	na	na	na
Morales-Gómez et al., 2020	2	2	1	2	2	2	2	0	na	na	na	na
Yan et al., 2023	2	0	1	2	2	2	0	0	na	na	na	na
Dobran et al., 2022	2	2	1	2	2	2	0	0	na	na	na	na
Guzel et al., 2008	2	1	2	2	2	2	1	0	na	na	na	na
Hashimoto et al., 2023	2	2	1	2	2	2	0	0	na	na	na	na
Honda et al., 2021	2	0	1	2	2	2	0	0	na	na	na	na
Mersha et al., 2020	2	0	1	2	2	2	2	0	na	na	na	na
Ozdol et al., 2024	2	0	1	2	2	2	0	0	na	na	na	na
Sauvigny et al., 2024	2	0	1	2	2	2	1	0	na	na	na	na
Tabuchi et al., 2014	2	2	1	2	2	2	1	0	na	na	na	na

## Discussion

Through comprehensive literature review aggregating evidence regarding LA and GA for cSDH evacuation, we found that LA resulted in lower surgery duration and postoperative hospital stay as well as more favorable complication rates. LA did not show any advantage in terms of recurrence, mortality, or wound infection rates. Overall, studies underlined the safety of cSDH evacuation in local anesthesia, especially regarding potentially protracted postoperative courses. Of note, most patients were of advanced age and presented with multiple comorbidities, most commonly including arterial hypertension, cardiovascular and psychiatric conditions, constituting significant risk factors for general anesthesia [49]. However, quantitative comparison of risk factors between groups, e.g. concerning antithrombotic drug intake, was limited by differential reporting across studies. This caveat further underlines the necessity for future prospective trials with detailed reporting.

Recent reviews by Abdelhady et al*.* [1] and Liu et al*.* [35] have already summarized the current literature on LA versus GA in cSDH evacuation, building on Liu et al*.’*s 2014 review [34] of these two anesthesiologic approaches in this context. However, these reviews grouped different surgical techniques which vary significantly in their invasiveness and perioperative risk, thereby influencing anesthesia duration and postoperative complications. In contrast to these reviews, we aimed to further refine the inclusion criteria, focusing exclusively on studies examining burr hole evacuation as the surgical technique. The rationale for this review is to specifically provide evidence supporting this widely used and well-established intervention, which represents the most common approach for treating cSDH [33]. Overall, our findings align with the previous conclusions of Abdelhady et al*.* [1] and Liu et al*.* [35]*,* particularly in confirming the safety and feasibility of surgery under LA.

Consistent with existing literature, our findings indicate that LA is associated with a shorter surgery duration. Notably, surgical techniques were uniform across groups, ensuring that our results were not influenced by variations in procedure duration – a limitation seen in previous reviews that included both craniotomies and burr hole evacuations [1, 35]. However, periprocedural tasks such as patient positioning and immediate postoperative care are likely more efficient in LA, as patients remain awake, cooperate, and do not require extubation. These factors are often incorporated into surgery or in-theater times as proxies for resource utilization. Given the heterogeneous and incomplete reporting in the included studies, future research with a more detailed breakdown of procedural times is needed to provide greater context to this finding.

Another important critique of previous literature cited possible bias in included studies [41]. To address this issue, we opted to include descriptive data of single-arm reports that are less likely to be confounded towards LA or GA. Here, we found that both underlying demographics as well as complication rates and outcome measures were largely similar between single-arm studies and the LA and GA arms of comparative studies. In an additional subanalysis, we further limited studies to those that confirmed usage of subdural drainage, where we found significantly shorter surgery duration and hospital stays associated with LA. While the direction of risk ratios remained similar, statistical significance was reached only for complication rates, potentially due to a scarcity of underlying data. Taken together, while warranting further confirmation in prospective trials, LA remained favorable to GA in this subanalysis.

The safety and feasibility of LA in surgery for elderly patients have been well established in previous literature, even extending to neurosurgical indications. There is growing evidence for the benefit of LA over GA in terms of complication rate and mortality, including in spine surgery [30, 46] and arthroplasty [36, 43]. On the other hand, GA offers advantages in terms of airway control and allows for conversion to craniotomy [15, 49]. However, none of the included studies mentioned the occurrence of such transitions, and the potential benefits of GA were not mirrored in statistically relevant effects in this review. In an aging population necessitating surgical interventions for degenerative and age-related conditions, LA is increasingly recognized as a viable alternative to GA. However, current literature lacks evidence on potential benefits concerning the occurrence of postoperative delirium, particularly in elderly patients. This is, in part, due to the retrospective nature of most reports in this field. In the current review, delirium was not reliably and systematically assessed in any included study using standardized delirium assessment tools. However, given the high prevalence and disease burden of delirium, it is of high clinical relevance and should be reported especially in studies collecting evidence for an outstandingly vulnerable patient group [55]. This further underlines the imperative to conduct prospective, randomized trials with validated delirium assessment tools [2].

### Strength and limitations

By the nature of retrospective studies, there are limitations about the comparability and potential biases concerning group allocation. Descriptions of the allocation criteria and process remained opaque in most studies, thus establishing the need of future prospective randomized trials to ensure the generalizability of prior findings. Similarly, reporting of anesthetic protocols, preoperative GCS and ASA scores was limited and significantly heterogeneous between studies.

Antithrombotic medication poses a significant risk factor for cSDH formation and postoperative complications [9, 11, 26]. Of note, studies included in this review did not report if or how antithrombotic agents were antagonized prior to surgery. Three studies, namely, Morales-Gómez et al*.,* Yan et al*.,* and Hashimoto et al*.,* mentioned temporary discontinuation of any antithrombotic medication [20, 40, 62]. In future trials, this information needs to be included to avoid confounding of recurrence and complication measures.

One decisive restriction in the current literature is the lack of studies specifically studying local anesthesia without additional sedation, thus not accurately representing on the feasibility of cSDH surgery in LA alone. Alike hypnotics used in GA, sedatives can cause delirium, disorientation and delayed recovery. As such, surgery in infiltrative LA without further sedation, upon establishment of its safety, might be the most beneficial in patients susceptible for GA complications. Within the studies included in this review, only Zhuang et al*.* reported no additional sedation of patients in the LA group [63]. Our results therefore might underestimate the benefits of LA concerning recovery times, reflected as length of hospital stay in this review. This deficit among others warrants further studies specifically investigating LA without sedation to deliver reliable data on the safety and efficacy of LA.

Overall, the studies included in the current review revealed low heterogeneity (I^2^) measures, however, regarding surgery duration and postoperative hospital stay, we found high heterogeneity, impacting the generalizability of some of our pooled results. These factors might also be influenced by site-specific differences in data acquisition and clinical protocols. To showcase these variations, statistics were plotted from singular studies (Figs. 2–4**).** However, this heterogeneity in the present literature further underscores the need for prospective randomized studies to ensure robust and generalizable results, and to identify potential benefits and risk of cSDH surgery in LA vs GA with greater confidence.

## Conclusion

In this systematic review and meta-analysis, we found that LA was linked to lower overall complications, shorter operative time and postoperative hospital stay, while recurrence and mortality were similar in both groups. In total, the existing literature is heterogeneous and provides preliminary support for the safety and feasibility of awake surgery for cSDH in LA. Further research, including prospective randomized trials, is needed to comprehensively evaluate the safety and benefits of cSDH surgery under LA, with particular emphasis on its potential impact on postoperative delirium incidence.

## Data Availability

No datasets were generated or analysed during the current study.

## Abbreviations

aSDH: Acute subdural hematoma
ASA: American Association of Anesthesiologists classification
cSDH: Chronic subdural hematoma
CI: Confidence interval
DOAC: Direct oral anticoagulants
FDR: False discover rate correction
GA: General anesthesia
LA: Local anesthesia
MD: Mean difference
MINORS: Methodological index for non-randomized studies
RE: Random effect model
PRISMA: Preferred Reporting Items for Systematic Reviews and Meta-Analyses
RR: Risk ratio
SD: Standard deviation
SE: Standard error
VKA: Vitamin K antagonists
